# Supplementary material for: On the performance improvement of Butterfly Optimization approaches for global optimization and Feature Selection
Source: PLoS One. 2021 Jan 8;16(1):e0242612. doi: 10.1371/journal.pone.0242612 (PMC7793310; doi:10.1371/journal.pone.0242612)
Supplement: S1 File — (DOCX) [file pone.0242612.s003.docx]

1. 3 different approaches have been introduced to enhance Butterfly Optimization Algorithm. Based on chaotic local search and opposition based learning
2. 30 functions has been used to test these algorithms and a fair comparison has been done with number of algorithms
3. 5 datasets have been used to test and prove the efficiency of our algorithms
