# Supplementary material for: On the performance improvement of Butterfly Optimization approaches for global optimization and Feature Selection
Source: PLoS One. 2021 Jan 8;16(1):e0242612. doi: 10.1371/journal.pone.0242612 (PMC7793310; doi:10.1371/journal.pone.0242612)
Supplement: Attachment — Submitted filename: Response to Reviewers.docx [file pone.0242612.s004.docx]

We would like to thank the editors and reviewers for careful and thorough reading of this manuscript and for the thoughtful comments and constructive suggestions, which help to improve the quality of this manuscript. Our response follows:

**list of Responses to the Reviewer’s Comments**

| **Reviewer #1** | |
| --- | --- |
| **Reviewer Comments** | **Authors Responses** |
| This is a good work, but a number of major and minor amendments are required as follows:   1. Potential applications of the proposed CLSOBBOA should be discussed 2. There is no justification of the CLSOBBOA method. Why for this problem area, please discuss. There are many other similar methods in the literature in this area, so such a justification is required. 3. There is no statistical test to judge about the significance of the CLSOBBOA’s results. Without such a statistical test, the conclusion cannot be supported. 4. There is no discussion on the cost effectiveness of the proposed CLSOBBOA method. What is the computational complexity? What is the runtime? Please include such discussions. You can also use the big oh notation to show the computation complexity. 5. To have an unbiased view in the paper, there should be some discussions on the limitations of the proposed CLSOBBOA method 6. Analysis of the results is missing in the paper. There is a big gap between the results and conclusion. There should be the result analysis between these two sections. 7. How do you ensure that the comparison between CLSOBBOA and the comparative methods is fair? 8. The proposed CLSOBBOA method might be sensitive to the values of its main controlling parameter. How did you tune the parameters? 9. Avoid using first person. 10. Highlights are missing. 11. Avoid using abbreviations and acronyms in title, abstract, headings and highlights. 12. Please avoid having heading after heading with nothing in between, either merge your headings or provide a small paragraph in between. | **All the comments and suggestions have been considered, answered and new paragraphs have been added to the paper as follows.**   1. **Reply:** CLSOBBOA has been already applied for FS as in Page 26-28. 2. **Reply:** illustration statement has been added into the introduction to shows the problem of the original BOA as shown in page 2. 3. **Reply:** Wilicoxon Rank Sum have been used as shown in page 9. 4. **Reply:** CLSOBBOA complexity has been calculated as shown in page 3 and 6. 5. **Reply:** As stated in the manuscript. There is no algorithms can solve all optimization problems according to NFL as shown in page 2. 6. **Reply:** Results section has been modified and enhanced as in page 6-8. 7. **Reply:** as stated in the manuscript all algorithms has been executed on the same machine and repeated for 20 times 8. **Reply:** The Original paper argued that BOA parameter is selected after many experiments 9. **Reply:** All manuscript has been modified 10. **Reply:** Highlights has been added in separate file 11. **Reply:** All manuscript has been modified 12. **Reply:** some sentences have bben added between titles |

| **Reviewer #2** | |
| --- | --- |
| **Reviewer Comments** | **Authors Responses** |
| The author have introduced three improved versions of BOA to prevent the original algorithm from getting trapped in local optima and have a good balance between exploration and exploitation abilities. However, there are few points have been raised though out the review that need to be addressed by the author as below.   1. Potential applications of the proposed method should be discussed 2. There is no justification of the method. Why for this problem area, please discuss. There are many other similar methods in the literature in this area, so such a justification is required. 3. There is no statistical test to judge about the significance of the method’s results. Without such a statistical test, the conclusion cannot be supported.  4. There is no discussion on the cost effectiveness of the proposed method. What is the computational complexity? What is the runtime? Please include such discussions. You can also use the big oh notation to show the computation complexity.  5. To have an unbiased view in the paper, there should be some discussions on the limitations of the proposed method  6. The proposed method might be sensitive to the values of its main controlling parameter. How did you tune the parameters?  7. The given discussion on the obtained results should be further improved | **All the comments and suggestions have been considered, answered and new paragraphs have been added to the paper as follows.**   1. **Reply:** CLSOBBOA has been already applied for FS as in Page 26-28. 2. **Reply:** illustration statement has been added into the introduction to shows the problem of the original BOA as shown in page 2. 3. **Reply:**  Wilicoxon Rank Sum have been used as shown in page 9. 4. **Reply:** CLSOBBOA complexity has been calculated as shown in page 3 and 6. 5. **Reply:**  As stated in the manuscript. There is no algorithms can solve all optimization problems according to NFL as shown in page 2. 6. **Reply:** The Original paper argued that BOA parameter is selected after many experiments      7. **Reply:** discussion section have been updated as in page 6-8. |
